# Interpatient Variability in Dexmedetomidine Response: A Survey of the Literature

**DOI:** 10.1155/2014/805013

**Published:** 2014-01-16

**Authors:** Samantha F. Holliday, Sandra L. Kane-Gill, Philip E. Empey, Mitchell S. Buckley, Pamela L. Smithburger

**Affiliations:** ^1^University of Pittsburgh School of Pharmacy, 3501 Terrace Street, Pittsburgh, PA 15261, USA; ^2^Department of Pharmacy and Therapeutics, University of Pittsburgh School of Pharmacy, 3501 Terrace Street, Pittsburgh, PA 15261, USA; ^3^Banner Good Samaritan Medical Center, Department of Pharmacy, 1111 E. McDowell Road, Phoenix, AZ 85006, USA

## Abstract

Fifty-five thousand patients are cared for in the intensive care unit (ICU) daily with sedation utilized to reduce anxiety and agitation while optimizing comfort. The Society of Critical Care Medicine (SCCM) released updated guidelines for management of pain, agitation, and delirium in the ICU and recommended nonbenzodiazepines, such as dexmedetomidine and propofol, as first line sedation agents. Dexmedetomidine, an alpha-2 agonist, offers many benefits yet its use is mired by the inability to consistently achieve sedation goals. Three hypotheses including patient traits/characteristics, pharmacokinetics in critically ill patients, and clinically relevant genetic polymorphisms that could affect dexmedetomidine response are presented. Studies in patient traits have yielded conflicting results regarding the role of race yet suggest that dexmedetomidine may produce more consistent results in less critically ill patients and with home antidepressant use. Pharmacokinetics of critically ill patients are reported as similar to healthy individuals yet wide, unexplained interpatient variability in dexmedetomidine serum levels exist. Genetic polymorphisms in both metabolism and receptor response have been evaluated in few studies, and the results remain inconclusive. To fully understand the role of dexmedetomidine, it is vital to further evaluate what prompts such marked interpatient variability in critically ill patients.

## 1. Introduction

Five million patients are admitted to the intensive care unit (ICU) in the United States annually, and over 55,000 patients are cared for daily [1]. The number one reason for admission is respiratory failure/insufficiency, which often necessitates mechanical ventilation with the need of sedative medications [1]. Sedation in the ICU is utilized to optimize patient care by reducing anxiety, reducing agitation, and maximizing patient comfort and safety [2–8]. Previously, heavily sedating patients was a common practice in the ICU with the belief that it would help patients tolerate mechanical ventilation; however, currently, lighter sedation has been emphasized [9].

Oversedation is associated with increased time on the mechanical ventilator and length of stay in the ICU [9–11]. However, undersedation can be associated with severe patient agitation resulting in longer durations of ICU stay, time on mechanical ventilation, physiological stress, and increased rates of self-extubation [12, 13]. Undersedation may also result in hypercatabolism, suppressed immune system, and increased sympatholysis [9]. Achieving a balance between the two extremes is vital to optimize patient care.

In 2013, the Society of Critical Care Medicine (SCCM) released updates to the guidelines for management of pain, agitation, and delirium in the ICU. Lighter sedation coupled with daily sedation awakenings and measurement of sedation levels was emphasized. Opioids followed by non-benzodiazepines such as dexmedetomidine and propofol were recommended as first line agents for analgosedation [11]. Compared to alternative sedatives, benzodiazepines have been associated with increased time to liberation from the mechanical ventilator, hospitalization days, and risk of developing ICU-related delirium [11, 14–16].

Dexmedetomidine is a potent alpha-2 agonist and up to 8 times more selective than clonidine, an alpha agonist, for the alpha-2 receptor [17–25]. In contrast to benzodiazepines, recent large clinical trials have highlighted the benefits of dexmedetomidine including more delirium-free and coma-free days [4], a lighter sedation described as “cooperative sedation,” [20–31] and an opioid sparing analgesia with no added respiratory depression [18–25, 27–30, 32–38]. Both propofol and dexmedetomidine offer the advantage of easy titration and a relatively short half-life [11]. These non-benzodiazepine sedatives are not without concerns for adverse effects including hypotension and bradycardia [7].

Although dexmedetomidine use has increased over the years, its role in clinical practice is mired by observations that the ability to achieve sedation goals is not consistent amongst all patients. In the MIDEX and PRODEX trials, Jakob and colleagues noted that there was a lack of efficacy in at least 1 out of 8 patients [7]. Other studies have noted failure rates as high as 21% and 50% [8, 39]. The reasons for the wide variability in clinical response are unclear; however, several hypotheses have been considered. This paper reviews the available data regarding the role of patient features/traits, the pharmacokinetic differences present in critically ill patients, and the specific contribution of genetic polymorphisms to altered pharmacokinetics and pharmacodynamics.

## 2. Individual Patient Features/Traits 

The first hypothesis to be considered is that there are individual patient differences, not solely explained by genetic differences or pharmacokinetics, that produce the interpatient variability in response to dexmedetomidine (Table 1). Several studies have been conducted analyzing race, gender, and other unique characteristics with conflicting results. A retrospective study of 85 dexmedetomidine infusions in critically ill patients found that non-Black race was associated with a higher rate of intolerance/failure (odds ratio of 9.5; 95% CI 1.16–77.91, *P* = 0.03) and a reported intolerance/failure rate of 21% [8]. However, a prospective observational study in 73 healthy individuals found no association between race and intolerance/failure (*P* > 0.16) [40]. Genetic differences are linked to ethnicities and may explain the different response rates seen in the retrospective study [41–45]. Since both population sizes were small, it is difficult to ascertain the true relationship.

Three pharmacokinetic studies (Table 2) analyzed several covariates in the pharmacokinetic models. The first study in 13 patients showed that in patients with a lower baseline Simplified Acute Physiology Score (SAPS II), clearance of dexmedetomidine was higher and half-life shorter [46]. In the second study of 21 patients, a multivariate analysis found that dexmedetomidine clearance decreased with decreasing cardiac output and increasing age. Patients with low albumin concentrations also had an increased volume of distribution [47]; this was supported by a third study of 527 patients [48]. However, the third study also found no correlation between dexmedetomidine clearance and cardiac output or age. Only body weight was considered a strong predictor of dexmedetomidine clearance [48].

Smithburger and colleagues conducted a six month prospective, observational study evaluating dexmedetomidine effectiveness and if any variables were associated with drug response. Thirty-eight mechanically ventilated patients were analyzed. Thirty-two patients were Caucasian, and 19 patients were female. Overall, dexmedetomidine was ineffective in 19 (50%) of patients and effective in 11 (28.95%). The remaining eight cases (21%) were unable to be analyzed due to confounding variables affecting mental status. A lower Modified Acute Physiology and Chronic Health Evaluation (APACHE II) score and home antidepressant use were identified as significant factors for effective sedation with dexmedetomidine. Patients with higher APACHE II scores may require higher levels of sedation [39].

Other studies support the finding that dexmedetomidine should be used in patients requiring lighter sedation [3, 6]. Less information, however, is available about the interaction between home antidepressant use and alpha-2 agonists. It has been noted that antagonism of alpha receptors can decrease the time to effect for antidepressants while it is suggested that activation of alpha receptors may increase the time to effect [49, 50]. New antidepressants and antipsychotics have also been evaluated for their efficacy as alpha-2 receptors and antagonists [51, 52]. The interaction between dexmedetomidine and antidepressants, however, has not been fully understood and more research into underlying interactions and clinical impact is needed.

Overall, there is limited information about patient features or traits affecting response to dexmedetomidine. The two small studies that evaluated the role of race in response to dexmedetomidine reported opposite results. Few studies have identified covariates that may explain the wide range of interpatient variability. Hopefully, larger studies will take results from these pilot studies, such as from Smithburger and colleagues, and attempt to evaluate their validity.

## 3. Pharmacokinetics of Critically Ill Patients

The second hypothesis to be considered is that the variability in dexmedetomidine response can be explained by differences in the drug pharmacokinetics in critically ill individuals. Although the pharmacokinetics of healthy and postoperative patients have been evaluated, little data are present for critically ill patients. In healthy volunteers, dexmedetomidine has a linear, dose-independent pharmacokinetic profile [20, 21, 23–25, 30, 53]. It rapidly distributes in 6 minutes [18–22, 24, 25, 28, 30], is highly protein bound [19–25, 28, 30], and has a volume of distribution at steady state of 1.33 L/kg [19, 22, 28] or 118 L [20, 21, 24, 25]. Dexmedetomidine is almost completely metabolized in the liver by cytochrome P450 (CYP) enzyme 2A6 and uridine diphosphate glucuronosyltransferase (UGTs) glucuronidation pathways, specifically UGT1A4 and UGT2B10, to inactive metabolites [19–25, 28, 54]. Its estimated clearance is 39 L/hr [25, 30], and the elimination half-life is 2–2.5 hours [18–22, 24, 25, 28, 30]. Pharmacokinetics of dexmedetomidine do not vary with age, gender, or renal dysfunction in healthy volunteers [20–22, 25, 30, 55].

It is vital to investigate pharmacokinetics of dexmedetomidine in critically ill patients because evidence from healthy individuals demonstrates a dose-dependent response for sedation [53, 56–59]. A study demonstrated an increased sedation of 38% and 65% with the first two doses of dexmedetomidine that reached a targeted plasma level of 0.5 ng/mL and 0.8 ng/mL, respectively, in healthy individuals [57]. It is believed that the therapeutic range is up to 1.2 ng/mL but higher levels may be tolerated [60]. The relationship between dexmedetomidine concentrations and sedation levels is described as exponential (exponent < 1). The highest plasma concentrations (1.23 ± 0.29 ng/mL) increased cognitive performance time by a factor of two in healthy individuals [56].

Critical illness is known to alter drug pharmacokinetics [61]. End-organ damage and changes in hemodynamics, electrolytes, and hormones may alter medication dose-response relationships [17, 48, 62]. A study completed in postoperative patients suggested that pharmacokinetics remained similar to healthy individuals [63]. Three studies in critically ill patients suggest a wide variability in dexmedetomidine pharmacokinetics (Table 2) [46–48].

Iirola and colleagues examined the pharmacokinetics of long-term (>24 hour) high dose dexmedetomidine in 13 critically ill, Caucasian patients. They concluded that although the mean elimination half-life and clearance remained similar to those reported in healthy individuals, there was wide interpatient variability [46]. A second study by Iirola and colleagues examined the pharmacokinetics of prolonged (>48 hours) dexmedetomidine infusions in 21 critically ill patients and reported values similar to the first study. The elimination clearance (95% confidence interval) was 57 L/hr (42.1, 65.6), and volume of distribution at steady state was 132 L (96, 189) [47]. A third, larger study examined pharmacokinetics of prolonged (>24 hours) dexmedetomidine infusions in 527 patients and concluded that pharmacokinetics of the critically ill remained similar to healthy individuals. Again, there was very wide interpatient variability (coefficients of variation of approximately 60% for both volume of distribution and clearance) that was not fully explained by covariates. The strongest predictor of dexmedetomidine clearance was body weight [48].

As the three pharmacokinetic studies showed wide interpatient variability in dexmedetomidine plasma levels, it is important to question if adequate plasma levels are reached in critically ill patients. Recent trials have used higher doses (>0.7 mcg/kg/hr) to obtain desired sedation levels [4, 5, 7, 32, 64]. This suggests that adequate plasma levels have not been obtained; however, a retrospective study concluded that critically ill patients treated with high dose dexmedetomidine (doses > 0.7 mcg/kg/hr) had more Richmond Agitation Sedation Scale (RASS) scores outside of targeted sedation than those treated with low dose (doses ≤ 0.7 mcg/kg/hr). They did not collect plasma levels [65]. Since the plasma level studies were completed in healthy individuals and the retrospective study was completed in a small population without plasma levels, it is difficult to ascertain if appropriate plasma levels were reached. More studies are needed to evaluate if a goal dexmedetomidine level is ideal and if it is being achieved in critically ill patients.

## 4. Genetic Polymorphisms

### 4.1. Metabolism

The third hypothesis to explain the variability of response to dexmedetomidine is that there are clinically-relevant genetic polymorphisms in metabolism. Dexmedetomidine is metabolized by CYP2A6, which is also responsible for the metabolism of coumarin, valproic acid, and nicotine. CYP2A6 can be induced by phenobarbital and dexamethasone [66]. Several polymorphisms with significant interindividual and interracial variations that affect drug metabolism have been reported [41, 66–68]. A meta-analysis by Carter and colleagues reported that CYP2A6*2 was more common in individuals of European descent (1–3%) and rare in the Asian population. In contrast, the polymorphism CYP2A6*4 was more common in individuals of Asian descent (15–20%) and rare in Caucasian populations [41, 42]. Both alleles are associated with poor metabolism [42, 67, 68]. Due to the paucity of substrates, the importance of polymorphisms in CYP2A6 is not well understood. However, this may contribute to dexmedetomidine's clinical response variability [42]. Two studies have evaluated the role of CYP2A6 polymorphisms in dexmedetomidine metabolism (Table 3).

Kohli and colleagues examined the role of polymorphisms of CYP2A6 on dexmedetomidine disposition using a Bayesian hierarchical mixture pharmacokinetic model of dexmedetomidine blood levels. Their investigation included 43 mechanically-ventilated patients that were enrolled in the MENDS trial and were being cared for in the medical and surgical ICUs [4]. This patient population consisted primarily of Caucasian individuals, accounting for 38 of the 43 enrolled, and five Black individuals. Patients were grouped into three categories: normal metabolizers (*n* = 33), intermediate metabolizers (*n* = 5), or slow metabolizers (*n* = 2). The authors concluded that clearance was not altered significantly by differences in polymorphisms of CYP2A6 [69]. A second study incorporated CYP2A6 pharmacogenomic genotypes into a population pharmacokinetic model and found no improvement in the goodness of fit [62].

Both studies determined that there was no significant difference or no effect of polymorphisms on dexmedetomidine metabolism, but these studies suffer from a small patient population size and lack of ethnic diversity. Both studies included only 43 patients with little to no representation of Asian ethnicity, where much variability is found [62, 69]. In order to have a more comprehensive picture of the effects of CYP2A6, a larger, more diverse population size is needed.

A second route of metabolism for dexmedetomidine is through glucuronidation by UGTs [54, 74]. Less is known about the importance and the variability in UGTs than the cytochrome P450 enzymes [75]. However, it has been demonstrated that there are polymorphisms in phase II metabolism, specifically UGTs and that these differences may affect drug metabolism [74–81].

Dexmedetomidine is metabolized by UGT1A4 and UGT2B10 [54, 74]. These enzymes are primarily involved in N-glucuronidation of endogenous estrogens and eicosanoids and of exogenous antidepressants and nicotine [74, 75]. Polymorphic differences have been implicated in patient intervariability for lamotrigine [79], valproic acid [78], and olanzapine [80]. While several polymorphisms have been identified, only two, UGT1A4*2 and UGT1A4*3, have been identified as altering function of the enzyme. There is limited and conflicting data regarding the functional impact of these polymorphisms. For example, for UGT1A4*2, a study by Ehmer and colleagues noted decreased enzymatic activity for beta-naphthylamine [82]; yet Wiener and colleagues noted an increased enzymatic activity for 4-(methylnitrosamino)-1-(3-pyridyl)-1-butanol [83]. Like with CYP2A6, the polymorphism frequencies appear to be ethnically linked [77, 78].

The second enzyme, UGT2B10, is less understood than UGT1A4, and there are fewer studies evaluating possible effects of polymorphisms [84]. The enzyme was just recently discovered in 2007 [54, 74, 75, 84]. Berg and colleagues examined the effect of UGT2B10 polymorphisms, specifically the single nucleotide polymorphism (SNP) Asp67Tyr, on nicotine metabolism. They noted that the polymorphism Asp67Tyr was associated with skewed amounts of metabolites and with higher oxidative metabolites [81].

Dexmedetomidine is believed to be more extensively metabolized by UGT1A4 than UGT2B10 [54, 74]. The implications of this information are currently unknown as no studies have been done comparing polymorphic differences in glucuronidation metabolism in dexmedetomidine. Information is also lacking regarding cytochrome-mediated metabolism of dexmedetomidine. The only study to date was completed in a small group of patients with limited ethnic variability. More information in this area is desperately needed to grasp a fuller understanding of metabolism polymorphisms [75].

### 4.2. Pharmacodynamics

Genetic differences in each of the subtypes of the alpha-2 receptors may also affect patient response to dexmedetomidine [85]. Since an alpha subtype specific agent has not been identified, the specific roles of each of the receptors have been difficult to define. It is currently known that Alpha-2A (ADRA-2A) is responsible for suppressing sympathetic flow from the brain producing a decrease in blood pressure, heart rate, and cardiac output. It also produces antinociceptive, sedative, and anxiolytic effects [21, 23, 25, 28, 86, 87]. It also inhibits norepinephrine release from sympathetic nerve endings [86]. Alpha-2B (ADRA-2B) is responsible for vasoconstriction [22, 29, 72, 86]. Alpha 2-C (ADRA-2C) is responsible for mediating dopamine neurotransmission [86, 87].

If a genetic difference produced a receptor with a lower affinity to dexmedetomidine, then a higher concentration of dexmedetomidine would be necessary to produce the same response seen in a wild-type (wt) receptor. These genetic differences may also produce nonfunctioning receptors which would not produce a response from dexmedetomidine. In order to examine these polymorphisms, researchers have examined all three subtypes to see if polymorphisms could be responsible for large interpatient variability in response to dexmedetomidine (Table 3).

Two important polymorphisms have been identified for ADRA2A [88, 89]. The first genetic polymorphism is ADRA2A −1291C/G SNP [89]. This SNP has been investigated in attention deficit hyperactivity disorder (ADHD) as well as other behavioral conditions such as Tourette's [43, 90]. It has also been implicated in interpatient variability to medications such as weight gain in antipsychotics [91] and antidepressant response to milnacipran [92]. Ethnic differences have been identified for this polymorphism [43–45]. A second common polymorphism, C753G transversion, has not been studied in dexmedetomidine [88].

Yağar and colleagues examined the effects of ADRA2A C1291G polymorphism on dexmedetomidine response in 110 patients undergoing a coronary artery bypass graft (CABG). Patients were monitored with bispectral index (BIS) monitor and Ramsey Sedation Score (RSS) every five minutes; once RSS ≤2, dexmedetomidine 1.4 mcg/kg/hr IV was given. Thirty minutes after the beginning of the dexmedetomidine infusion, the BIS scores were higher and RSS scores lower for patients carrying the G allele. This indicated that the patients with the G allele were less sedated than those with the C allele, and this difference was statistically significant. There was no genetic difference for differences in blood pressure or heart rate response to dexmedetomidine [70].

The results of this study were supported by a prospective, observational study in 73 healthy individuals. It analyzed nine different ADRA2A variants and concluded that certain polymorphisms were associated with statistically significant differences (*P* = 0.007) in changes in blood pressure [45].

Although the main effects of dexmedetomidine have been attributed to ADRA2A, researchers have also examined the effects of polymorphisms in ADRA2C and ADRA2B. For ADRA2C, the deletion of the four amino acids 322–325 has been identified and associated with a decrease in function [40, 93–95]. For dexmedetomidine, a nonfunctioning ADRA2C would imply a decrease in the inhibition of norepinephrine release and spinal analgesia producing decreased effectiveness [86, 93–95]. A prospective, observational study of 73 healthy individuals, however, concluded that there was no change in response (*P* > 0.66) for all genotypes [40]. This study was supported by a second prospective, observational study that found no difference in analgesic effects of dexmedetomidine in patients with wild-type and del322-325 ADRA2C [71].

The receptor ADRA-2B, mainly responsible for constriction, has also been evaluated for genetic polymorphisms to see if these could be implicated in interpatient variability [72, 86]. For ADRA-2B, one important deletion variation, del301-303, results in a desensitized receptor [72, 85, 96–101]. If a patient had a desensitized receptor, then it is thought that he/she would not respond as easily to plasma catecholamines. Since dexmedetomidine can inhibit norepinephrine release, less catecholamine could result in less vasoconstriction and more hypotension [18, 23, 24, 26, 37, 38].

Muszkat and colleagues examined the effect of the ADRA2B del301-303 receptor (del) and the wt receptor on peripheral vasoconstriction. They hypothesized that there would be a difference in venoconstriction response between wt and del receptors when dexmedetomidine was administered; however, they determined that venoconstriction response of both wt and del receptors was similar [72]. Muszkat and colleagues also evaluated the possibility that a pathway downstream from the receptor, independent of genetic variation, could be responsible. They reasoned that if factors such as concentration of the drug at the active site or effects downstream from the receptor are plausible explanations, then it is reasonable to assume that sensitivity to both alpha-1 and alpha-2 receptors is correlated [73, 102]. Muszkat and colleagues studied the effects of phenylephrine, an alpha-1 receptor agonist, and dexmedetomidine on venoconstriction in the dorsal hand vein. They determined that there was no significant correlation for venoconstriction response to dexmedetomidine. Muszkat and colleagues concluded that alpha-1 receptors and alpha-2 receptors are mediated differentially [73]. In a similar study, Posti and colleagues studied the venoconstriction of phenylephrine and dexmedetomidine in the dorsal hand model. They also concluded that the sensitivity and effects of each receptor are mediated separately [102].

More information concerning the differences in receptor polymorphisms has been gathered than in the differences in metabolism; however, understanding of the implications of the receptors' pharmacogenetics remains incomplete. Currently, studies have only been completed in small groups of individuals, and the results are confounded by the lack of a subtype-receptor specific agonist or antagonist. Research in other disease states such as ADHD has implicated polymorphisms of receptors as affecting response. This suggests that receptor polymorphisms are not yet well understood and may hold the key as to why there is certain interpatient variability. While all three receptor subtypes have been evaluated, ADRA-2A appears to be the primary receptor activated and responsible for effects. More studies evaluating receptor polymorphisms in ADRA-2A as well as the other two receptors are needed to determine if this is a plausible explanation.

## 5. Conclusion and Future Direction

It is difficult to characterize what prompts such marked interpatient variability in critically ill patients receiving dexmedetomidine. The few studies that have examined this phenomenon have not produced clinically significant answers to guide clinicians in appropriate patient selection or appropriate dosing. Both dexmedetomidine and propofol are recommended as first line sedatives in the SCCM guidelines. Although dexmedetomidine exhibits beneficial properties and may have some advantages over propofol, reports of treatment failure are concerning. Therefore, further research into individual patient features/traits, pharmacokinetics, and genetic polymorphisms, potentially impacting dexmedetomidine's efficacy, is vital to better define its role in therapy and to better optimize patient care.

## Figures and Tables

**Table 1:** Studies evaluating the role of patient features/traits.

Citation	Study design	Population	Objective	Intervention	Results
Tellor et al. (2012) [8]	R, O	(i) 75 MV adults with 85 episodes of DEX infusion. (ii) Included 50 Caucasian and 15 Black patients.	To describe rates of DEX intolerance/failure and identify patient predictors of intolerance/failure.	(i) All patients received DEX titrated by the bedside nurse from 0.2 mcg/kg/h to a maximum of 1.5 mcg/kg/h to achieve targeted sedation with rescue fentanyl, midazolam, and propofol permissible. (ii) Individual episodes of DEX infusions were analyzed for predefined descriptions of intolerance and failure. (iii) Multivariate logistic regression analysis was used to identify predictors of intolerance or failure.	(i) 18 episodes (21%) were classified as intolerance/failure. 67 episodes (79%) were classified as successful. (ii) No significant difference between success and intolerance groups in additional sedation use was noted. (iii) Non-Black race was found to be statistically significant (adjusted odds ratio 9.5; 95% CI 1.16–77.91; *P* = 0.03) as a predictor of failure/intolerance. The clinical significance of this was questionable.

Kurnik et al. (2008) [40]	P, O	(i) 73 healthy individuals. (ii) Included 37 Caucasian and 36 Black patients.	To evaluate the clinical effect of ethnicity on CV effects by changes in BP and HR and plasma NE concentrations.	(i) Patients received 3 placebo infusions then 3 DEX infusions (0.1, 0.15, 0.15 mcg/kg for total of 0.4 mcg/kg). (ii) BP, HR, and plasma NE concentrations were measured at the end of the infusion then 10 min and 20 min after. (iii) DEX plasma levels were measured after the last infusion of DEX. (iv) Patients were grouped into ADRA-2C del322-325 or no deletion and GNB3 C825T or no polymorphism.	(i) No significant differences in BP, HR, and plasma NE concentrations for ethnicity were detected. (i) Black patients had a higher plasma DEX concentration than white patients (mean ethnic difference 0.05 ng/mL; 95% CI, 0.03 to 0.07 ng/mL).

Smith-burger et al. (2013) [39]	P, O	38 MV patients (32 Caucasian, 6 Blacks).	To identify patient specific characteristics that affect achievement of successful sedation with DEX.	(i) All patients received DEX to achieve targeted sedation based upon unit sedation protocol. (ii) Doses ranged from 0.3 mcg/kg/hr to 1.4 mcg/kg/hr with rescue propofol, fentanyl, and benzodiazepines allowed. (iii) Individual episodes of DEX infusions were analyzed for predefined descriptions of intolerance and failure. (iv) Multivariate logistic regression analysis used to identify predictors of intolerance or failure.	(i) DEX was ineffective in 19 (50%) patients. It was effective in 11 (28.95%) patients. In the remaining patients (*n* = 8), an analysis was unable to be completed. (ii) No significant difference between success and intolerance groups in additional sedation use was noted. (iii) Lower APACHE II score (*β* coefficient −0.24; 95% CI, −0.39 to −0.03) and home antidepressant use (*β* coefficient 2.33; 95% CI, 0.23 to 4.43) were identified as positive patient specific characteristics.

R: retrospective; O: observational; P: prospective; MV: mechanically ventilated; DEX: dexmedetomidine; CV: cardiovascular; BP: blood pressure; HR: heart rate; NE: norepinephrine; ADRA-2C: alpha adrenergic receptor 2C; APACHE II: A Modified Acute Physiology and Chronic Health Evaluation.

**Table 2:** Studies evaluating pharmacokinetics in critically ill patients.

Citation	Study Design	Population	Objective	Intervention	Results
Iirola et al. (2011) [46]	P, O	(i) 13 ICU patients. (ii) All Caucasian.	To describe PK of prolonged (>24 hrs) and HD DEX in critically ill patients and to determine if a linear relationship remains.	(i) All patients received DEX at a constant rate for the first 12 hours (doses were 0.1, 0.2, 0.45, or 0.7 mcg/kg/hr with no LD) then titrated by bedside nurse to goal sedation score. The dose allowed was 0.1–2.5 mcg/kg/hr. (ii) DEX plasma levels were obtained at predefined times.	(i) DEX retained a linear relationship at doses of 2.5 mcg/kg/hr (*r* ^2^ = 0.95). (ii) PK parameters remained similar to those reported in healthy individuals. (iii) A multivariate analyses showed a significant difference in higher Cls (*P* = 0.006) and shorter elimination half-life (*P* = 0.036) with lower baseline SAPS II (<42) score.

Iirola et al. (2012) [47]	P, O	21 ICU patients.	To describe PK of prolonged (>48 hrs) DEX infusions in critically ill patients.	(i) All patients received a LD of 3–6 mcg/kg/hr over 10 min then a maintenance infusion of 0.1–2.5 mcg/kg/hr titrated by bedside nurse to desired sedation. (ii) DEX plasma levels were obtained at predefined times.	(i) PK parameters remained similar to those reported in healthy individuals. (ii) The elimination Cls was 57.0 L/hr (42.1, 65.6) and *V*_ss_ was 132 L (96, 189). (iii) A multivariate analyses showed that DEX Cls decreased with decreasing CO and increasing age (*P* = 0.009); *V*_ss_ was increased in patients with low albumin concentration (*P* = 0.002).

Välitalo et al. (2013) [48]	P, O	(i) 527 ICU patients enrolled in phase III studies of prolonged (>24 hrs) DEX infusion. (ii) 96% were Caucasian.	To describe PK of prolonged (>24 hrs) DEX infusions in critically ill patients.	(i) All patients received an initial infusion of 0.7 *µ*g/kg/h for 1 hour then a maintenance infusion of 0.2–1.4 *µ*g/kg/h titrated by bedside nurse to desired sedation. (ii) Maximum duration of infusion was 14 days. (iii) DEX plasma levels were obtained at baseline, 1 hr after beginning treatment, and then the same time each day. Samples were taken 24 and 48 hours after infusion ended.	(i) PK parameters remained similar to those reported in healthy individuals. (ii) The strongest covariate relationship was between DEX Cls and body weight. (iii) A multivariate analysis showed an inverse relationship between plasma albumin and *V*_ss_; however, this relationship did not account for interpatient variability. (iv) DEX Cls was not affected by CO or concentration levels.

P: prospective, O: observational; ICU: intensive care unit; PK: pharmacokinetics; HD: high dose; DEX: dexmedetomidine; LD: loading dose; Cls: clearance; SAPS II: Simplified Acute Physiology Score; CO: cardiac output; *V*_ss_: volume of distribution at steady state.

**Table 3:** Studies evaluating role of genetic polymorphisms.

Citation	Study design	Gene studied	Population	Objective	Intervention	Results
Kohli et al. (2012) [69]	P, O	CYP2A6	43 MV patients in medical and surgical ICU (38 Caucasian and 2 Black).	To determine the effect of CYP2A6 polymorphisms on DEX Cls.	(i) All patients received DEX. (ii) Patients grouped into normal (*n* = 33), intermediate (*n* = 5), or slow (*n* = 2) metabolizers. (iii) Cls determined via median of five plasma DEX concentrations.	No significant difference in DEX amongst normal (48.5 L/h; 39.8–58.7 L/hr), intermediate (56.9 L/hr; 31.6–94.4 L/hr) or slow (86.2 L/hr; 26.9–218.7 L/hr) metabolizers. Presented as (median; 95% credible interval)

Choi et al. (2011) [62]	P, O	CYP2A6	43 critically ill patients.	To develop a new population PK model to more accurately accommodate outliers.	(i) All patients received DEX titrated by the bedside nurse from 0.15 mcg/kg/h to a maximum of 1.5 mcg/kg/h to achieve targeted sedation. (ii) Serum levels were collected at three specific, predefined times (05:00 ± 2 h, 10:00 ± 2 hrs, 16:00 ± 2 h).	Incorporation of CYP2A6 genotype as a covariate did not alter the population pharmacokinetics.

Ya$\overset{ˇ}{\text{g}}$ar et al. (2011) [70]	P, O	ADRA-2A	110 patients undergoing CABG.	To evaluate the clinical effect of the ADRA-2A C-1291G polymorphism on DEX response via HD effects and sedation.	(i) All patients received DEX after surgery once in the ICU to a targeted infusion rate of 1.4 mcg/kg/hr. (ii) HD effects (SAP, DAP, HR) monitored via routine invasive monitoring. (iii) Sedation effects monitored via BIS and RSS. (iv) Patients were grouped into G allele carriers and noncarriers.	(i) No significant difference in HD (SAP, DAP, HR) effects. (ii) Patients with C-1291-G polymorphisms had higher BIS (*P* < 0.05) after 30 min and RSS (*P* < 0.05) after 55–60 min starting infusion, showing a longer period to fall asleep or a decrease in DEX effect.

Kurnik et al. (2011) [45]	P, O	ADRA-2A	73 healthy individuals.	To evaluate the clinical effect of ADRA-2A polymorphisms on CV effects of DEX by changes in SBP and plasma NE concentrations.	(i) Patients received 3 placebo infusions then 3 DEX infusions (0.1, 0.15, 0.15 mcg/kg for total of 0.4 mcg/kg). (ii) SBP and plasma NE concentrations were measured 10 min after infusion. (iii) Patients were grouped into 9 different polymorphisms.	(i) After a sensitivity analysis, individuals who were homozygous or carriers of HT3 had a 39% (0.61 fold) smaller decrease (ΔSBP= 8.8 ± 6.5 mmHG and 14.5 ± 6.1 mmHG, respectively; *P* = 0.015) in SBP after DEX. Individuals who were homozygous for HT4 had an 82% (1.8 fold) larger decrease (ΔSBP= 24.7 ± 8.1 mmHG compared to 13.6 ± 5.9 mmHG for non-carriers; *P* = 0.007). (ii) There was no difference in plasma NE levels.

Kurnik et al. (2008) [40]	P, O	ADRA-2C	73 healthy individuals (37 Caucasians, 36 Blacks).	To evaluate the presence of the ethnic differences in the response to DEX through the clinical effect of ADRA-2C del322-325 polymorphism and G-protein GNB3 C825T polymorphism on CV effects by changes in BP and HR and plasma NE concentrations.	(i) Patients received 3 placebo infusions then 3 DEX infusions (0.1, 0.15, 0.15 mcg/kg for total of 0.4 mcg/kg). (ii) BP, HR, and plasma NE concentrations were measured at the end of the infusion then 10 min and 20 min after. (iii) Patients were grouped into ADRA-2C del322-325 or no deletion and GNB3 C825T or no polymorphism.	No significant differences in BP, HR, and plasma NE concentrations for both ADRA-2C del322-325 and GNB3 C825T polymorphism were detected.

Kohli et al. (2010) [71]	P, O	ADRA-2C	73 healthy individuals (37 Caucasians, 36 Blacks).	To evaluate the clinical effect of several ADRA-2C polymorphisms on pain perception and cognitive responses.	(i) Pain rating via VAS from a cold pressor test before infusions and 30 min after the final DEX infusion. (ii) Cognitive responses were measured before the infusions, 10 min after the placebo and DEX infusions. (iii) Patients received 3 placebo infusions then 3 DEX infusions (0.1, 0.15, 0.15 mcg/kg for total of 0.4 mcg/kg). (iv) Patients were grouped into five different polymorphisms, including ADRA-2C del322-325.	(i) VAS scores from patients with del322-325 were significantly higher than any other polymorphism (*P* = 0.012). (ii) DEX lowered mean pain scores significantly (from 4.8 ± 2.1 cm to 4.1 ± 2.1, *P* < 0.001). (iii) The change in pain score after DEX administration was not significantly associated with any genotype. (iv) No genotype, especially del322-325, was significantly associated with differences in sedation scores or memory response.

Muszkat et al. (2005) [72]	P, O	ADRA-2B	49 healthy individuals.	To examine the clinical effect of ADRA-2B del 301-303 polymorphism on vascular response.	(i) Patients were grouped into wt/wt (*n* = 28), wt/del (*n* = 13), del/del (*n* = 8). (ii) Patients received DEX at increasing doses ranging from 0.01–1000 ng/min. (iii) Venoconstriction was measured via LVDT and the ED_50_ (dose that produced 50% of maximum venoconstriction or *E*_max_) was calculated. (iv) Maximum venoconstriction was the percentage of reduction in vein diameter from maximal dilation.	(i) There was no difference in ED_50_ and *E*_max_ among the three groups. (ii) The ED_50_ for wt/wt was 1.39 ng/min (95% CI 0.03–63 ng/min), for wt/del 1.63 ng/min (95% CI 0.01–177.8 ng/min), and for del/del 2.37 ng/min (95% CI 0.17–33.7 ng/min). (iii) The *E*_max_ for wt/wt was 75.4 ± 14.9%, for wt/del 75.7 ± 21.3%, for del/del 82.2 ± 12.9%.

Muszkat et al. (2011) [73]	P, O	*α*-1 and *α*-2	62 healthy individuals (28 Caucasian, 34 Blacks).	To determine if adrenergic vasoconstriction sensitivity through *α*1 and *α*2 receptors are determined through mechanisms downstream from the receptor and thus linked.	(i) Patients received either DEX at increasing doses ranging from 0.01–100 ng/min or phenylephrine at increasing doses ranging from 12–12000 ng/min. (ii) Venoconstriction was measured via LVDT and the ED_50_ (dose that produced 50% of maximum venoconstriction or *E*_max_) was calculated. (iii) Maximum venoconstriction was the percentage of reduction in vein diameter from maximal dilation.	(i) Median ED_50_ for DEX was 1.32 ng/min (IQR 0.45–5.37 ng/min) and for phenylephrine was 177.8 ng/min (IQR 40.7–436.5 ng/min). (ii) There was no correlation between ED_50 _(individual sensitivity) for DEX and phenylephrine before and after covariates. (iii) The *E*_max_ for DEX was 80.0% (64.7–95.2%) and for phenylephrine was 90.8% (82.2–99.6%).

P: prospective; O: observation; MV: mechanical ventilation, ICU: intensive care unit; DEX: dexmedetomidine; Cls: clearance; PK: pharmacokinetic; CABG: coronary artery bypass graft; ADRA-2A: alpha adrenergic receptor 2A; HD: hemodynamic; SAP: systolic arterial pressure; DAP: diastolic arterial pressure; HR: heart rate; ICU: intensive care unit; BIS: bispectral index; RSS: Ramsay Sedation Scale; CV: cardiovascular; SBP: systolic blood pressure; NE: norepinephrine; BP: blood pressure; VAS: visual analogue scale; IQR: interquartile range.
